# Case report: Plasma cell leukemia secondary to multiple myeloma successfully treated with anti-BCMA CAR-T cell therapy

**DOI:** 10.3389/fonc.2022.901266

**Published:** 2022-09-21

**Authors:** Jingjing Deng, Yuehui Lin, Defeng Zhao, Chunrong Tong, Alex H. Chang, Wenming Chen, Wen Gao

**Affiliations:** ^1^ Department of Hematology, Beijing Chaoyang Hospital, Capital Medical University, Beijing, China; ^2^ Department of Hematology, Beijing Boren Hospital, Beijing, China; ^3^ Clinical Translational Research Center, Shanghai Pulmonary Hospital, Tongji University School of Medicine, Shanghai, China; ^4^ Shanghai YaKe Biotechnology Ltd., Shanghai, China

**Keywords:** plasma cell leukemia, relapsed/refractory multiple myeloma, chimeric antigen receptor T cell therapy, secondary plasma cell leukemia, treatment

## Abstract

Chimeric antigen receptor (CAR)-T cell therapy provides an effective salvage treatment for relapsed/refractory multiple myeloma (RRMM) patients. End-stage RRMM with plasma cell leukemia (PCL) transformation is highly aggressive and resistant to conventional therapy. There is an urgent need for new therapeutics and CAR-T therapy may play an important role. We report a case of PCL secondary to RRMM successfully treated with CAR-T cell therapy targeting B-cell maturation antigen (BCMA). A woman was diagnosed as having MM 4 years ago and progressed to secondary PCL (sPCL) of five prior lines of treatment including proteasome inhibitors, an immunomodulatory agent, cytotoxic drugs, and an anti-CD38 monoclonal antibody. After receiving a BCMA CAR-T therapy, she achieved a stringent complete response that lasted 9 months. Then, the patient irregularly took venetoclax 10 mg per day due to a slightly higher λ FLC concentration, which did not meet the criteria for progression. She maintained a complete response for the following 7 months. In conclusion, BCMA CAR-T therapy may be a promising therapeutic approach in PCL patients. More studies are needed to evaluate the benefit of anti-BCMA CAR-T therapy in PCL patients.

**Clinical Trial Registration:** www.chictr.org.cn, ChiCTR1900024388, Registered 9 July 2019.

## Introduction

In recent years, the increasing prevalence of refractory/relapsed multiple myeloma (RRMM) shows resistance to conventional therapy (1). Therefore, developing new treatment strategies is an urgent need. Chimeric antigen receptor (CAR)-T therapy has become a groundbreaking approach in the treatment of multiple myeloma (MM). Nowadays, the Food and Drug Administration has approved idecabtagene vicleucel (Abecma, Bristol Myers Squibb) and ciltacabtagene autoleucel (CARVYKTI, Janssen Biotech, Inc.), which are both B-cell maturation antigen (BCMA)-directed genetically modified autologous CAR-T cell therapies, for the treatment of patients with RRMM after four or more prior lines of therapy, including a proteasome inhibitor (PI), an immunomodulatory agent (IMiD), and an anti-CD38 monoclonal antibody. However, MM may evolve to secondary plasma cell leukemia (sPCL), a highly aggressive end-stage dyscrasia with poor prognosis. Few data have been published about the potential benefit of CAR-T therapy in RRMM-transformed sPCL patients, specifically when leukemic symptoms are predominant. Here, we present a patient with RRMM who developed sPCL after five prior lines of treatment and was successfully treated with the anti-BCMA CAR-T therapy.

## Case presentation

**Figure 1:**
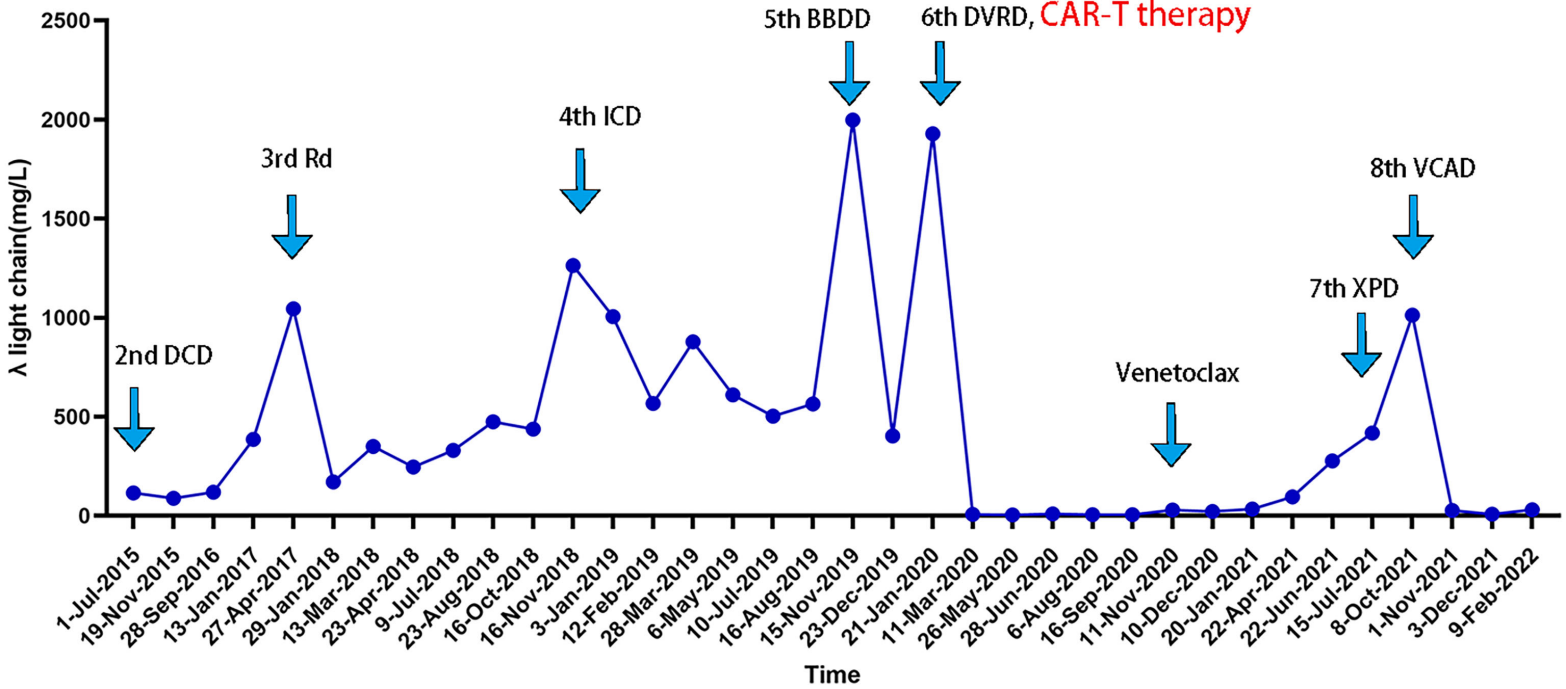
Free light chain concentration of the patient in different treatment stages.

A 60-year-old woman attended a local hospital with gum bleeding and back pain, and then underwent a series of laboratory tests and was diagnosed with IgG-λ MM [Durie-Salmon stage IIIA, International Staging System (ISS) stage III, Revised-ISS stage II]. She had been initially treated with four cycles of bortezomib-based therapy. Partial response (PR) was achieved after two cycles and sustained after two additional cycles of bortezomib-based therapy. Due to the failure of stem cell collection, she could not receive a planned autologous stem cell transplantation (ASCT). Additionally, the patient developed severe peripheral polyneuritis, which was thought to be an adverse effect of bortezomib. Hence, she was switched to four cycles of liposomal doxorubicin–cyclophosphamide–dexamethasone regimen. After 3 months, she maintained PR and received maintenance therapy. In April 2017, the disease progressed based on increasing λ free light chain (FLC) concentration. The patient was enrolled in a clinical trial and treated with Rd regimen (lenalidomide–dexamethasone), in which the best response was very good partial response (VGPR). In November 2018, the bone marrow (BM) smear revealed 68.5% plasma cells (PCs) with restrictive λ expression detected by flow cytometry (FCM). Her λ FLC concentration increased again. Considering the progression of the disease, she dropped out of the clinical trial and underwent regular chemotherapies with one cycle of ixazomib, cyclophosphamide, and dexamethasone (ICD) and followed by nine cycles of ICD regimen plus lenalidomide. In November 2019, BM biopsy revealed 81.5% PCs. There were 41% circulating plasma cells (CPCs) in the peripheral blood. Chromosomal karyotyping revealed 46,XX,t (1, 2)(q21;q33),add (2)(q33),t (3, 4)(q13;q32),add (5)(q24),del (6)(q12q22)[4]/46,XX[16]. Serum protein electrophoresis (SPEP) revealed a 0.3 g/dl M spike. The urinary light chain was quantified as 0.705 g per day. It was obvious that MM had progressed to sPCL. She was given a cycle of bendamustine–bortezomib–daratumumab–dexamethasone regimen. After that, there were 14.5% PCs in the bone marrow with 4% CPCs; SPEP showed a 0.3 g/dl M1 spike and a 0.1 g/dl M2 spike; the urinary light chain could not be quantified but immunofixation electrophoresis was positive, which showed that PR had been achieved. Then, another cycle of the daratumumab–bortezomib–dexamethasone regimen followed. Soon after, her λ FLC concentration increased to 1927.5 mg/L (**Figure 1**). BM smear revealed 68.5% PCs with 5% CPCs, which meant that the condition progressed again. All tumor cells expressed BCMA detected by FCM, indicating that there was sufficient BCMA expression on the monoclonal PCs, then CAR-T therapy became a key treatment. After half a cycle of the daratumumab–bortezomib–lenalidomide–dexamethasone regimen, the patient went to Beijing Boren Hospital for subsequent CAR-T therapy.

Preparation of BCMA CAR-T cells is as follows (2). The lentiviral vector was used to carry a second-generation BCMA-directed CAR with 4-1BB co-stimulatory and CD3ζ signaling domains provided by Shanghai YaKe Inc. After apheresis (circulating 5,000 ml of blood), the PBMCs were treated with lymphocyte separation liquid (GE Healthcare, Chicago, IL, USA) to remove granulocytes, red blood cells, and platelets. Briefly, PBMCs were stimulated with anti-CD3 and anti-CD28 monoclonal antibodies for 24 h and transduced with the lentivirus encoding murine anti-BCMA-CD3ζ-4-1BB CAR (from Shanghai Yake Biotech) and cultured for another 5 days. The next day, transduction was performed at a multiplicity of infection ratio of 1:5. The transduced cells were cultured in TexMACS Good Manufacturing Practices medium, containing 300 IU/ml IL-2 and 5 ng/ml IL-7, IL-15, and IL-21. The medium was replaced every 2 days. Transduction efficiency was 59.99%, which was defined as the ratio of CAR-T cells to CD3+ T cells determined using FCM and monoclonal antibody against BCMA (from Shanghai Yake Inc.).

**Figure 2:**
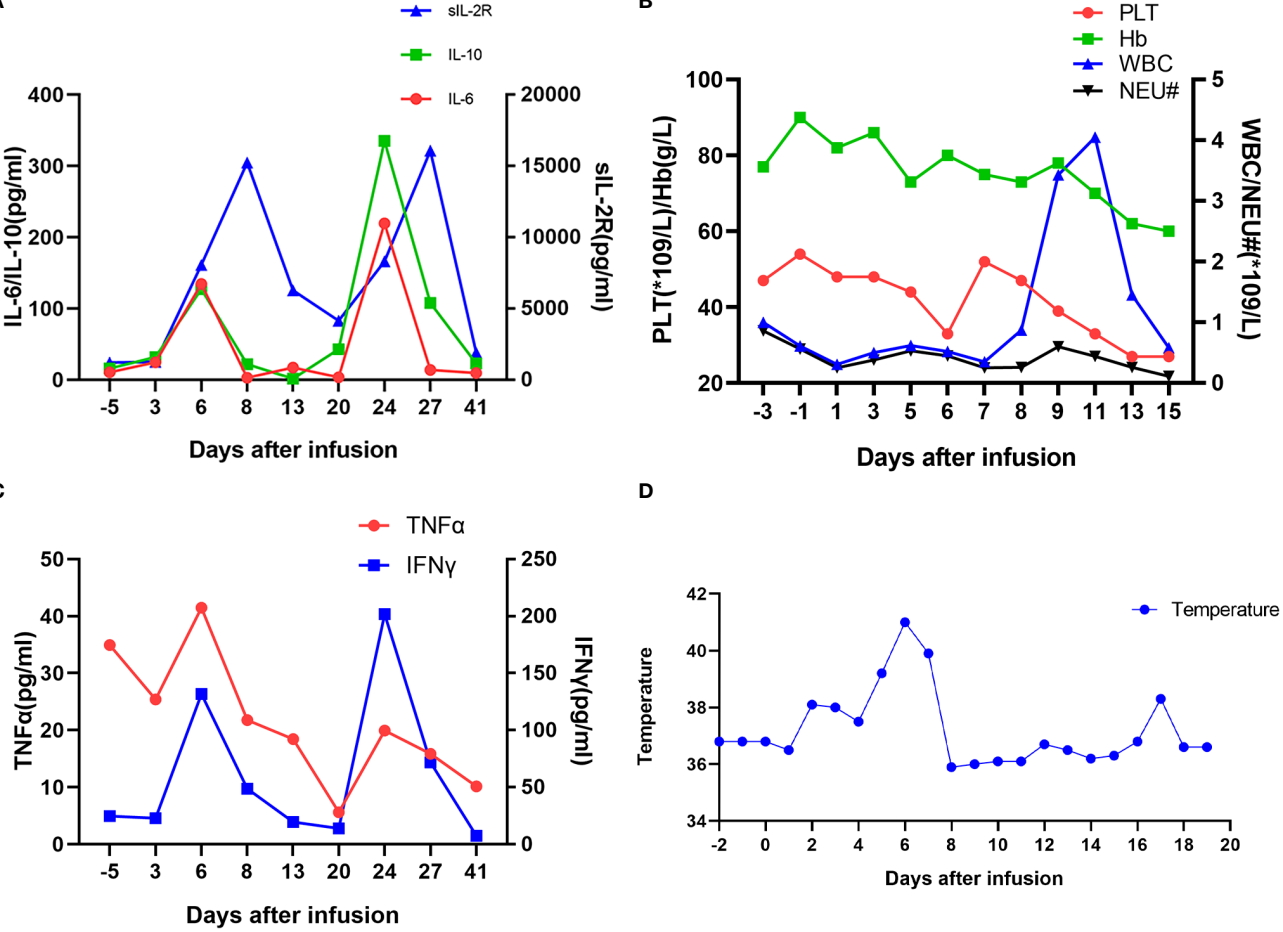
Measures of clinical responses to infusions of anti-BCMA CAR-T cells. **(A)** Concentrations of listed cytokines in serum obtained from the patient at the indicated time points. Day 0 is the day of anti-BCMA CAR-T cell infusion. **(B)** The results of blood routine tests and **(C)** serum TNFα and IFNγ concentrations are shown at the indicated time points after anti-BCMA CAR-T cell infusion. **(D)** The plot shows the changes of the maximum temperature for each day after anti-BCMA CAR-T cell infusion.

During the CAR-T cell manufacture, the patient had received conditioning chemotherapy with FC regimen (fludarabine 35 mg/m^2^ d1–3; cyclophosphamide 250 mg/m^2^ d1–3). Before CAR-T cells were infused, her blood cell counts were as follows: WBC was 0.61 × 10^9^/L; neutrophil, 0.56 × 10^9^/L; HGB, 90 g/L; PLT, 54 × 10^9^/L. She received administration of CAR-BCMA T cells (from Shanghai Yake Inc.) with the dose of 0.37 × 10^6^ CAR T cells/kg on 25 February 2020. The 19.6% CD3+ cells expressed BCMA CAR. Two days post-CART infusion, her blood cell counts were as follows: WBC 0.31 × 10^9^/L; neutrophil 0.25 × 10^9^/L. The patient had fever and became normal after ibuprofen suspension and meropenem, then had fever every day with the highest at 38.5°C. At 4 days post-CART infusion, she had oral ulcer and her blood tests were as follows: C-reactive protein (CRP) 11.23 mg/L; procalcitonin (PCT) 0.37 ng/ml. The acyclovir was infused. Six days post-CART infusion, she had a high fever and chills with a longer duration than previous days, with the highest temperature at 41°C, which does not go down even with 2 mg of dexamethasone infusion or ibuprofen suspension. The results of laboratory examination were as follows: WBC, 0.52 × 10^9^/L; neutrophil, 0.45 × 10^9^/L; CRP, 30.83 mg/L; PCT, 0.66 ng/ml; IL-6, 134.50 pg/ml; TNFα, 41.49 pg/ml; IL-10, 127.19 pg/ml; sCD25, 8,053 pg/ml; IFNγ, 131.80 pg/ml. To prevent severe cytokine releasing syndrome (CRS), a total of 17 mg of dexamethasone was infused. From Day 6 to Day 9, the patient had high fever and was relieved by glucocorticoids (**Figure 2**). On day 10 post-CART infusion, the patient’s temperature became normal. After CAR-T cell infusion, the patient only had a fever, which was not life-threatening, without hypotension, hypoxia, or grade 2 organ toxicity. The max grade of CRS was grade 1 according to the Lee criteria and the National Cancer Institute Common Terminology Criteria for Adverse Events version 5.0 (7). The CRS lasted about 9 days. However, approximately 20 days post-CART infusion, there was a second increase in the temperature, CRP, PCT, and cytokines, which mainly resulted from a suspected severe infection, probably mainly caused by bacteria. However, pathogenic identification and blood cultures were examined several times, with no result. The exact site of infection was not found. IVIG and stronger antibiotics tigecycline and aztreonam were separately applied.

The CAR-T cell expansion reached a peak of 53.00% on day 13 post-CART infusion, and the absolute CAR-T count was 3.90 × 10^8^/L. A high level of CAR-T cells (2.62 × 10^6^/L) was detected in peripheral blood on day 41 post-CART infusion, and CAR-T cells returned to baseline level 56 days after infusion.

Two weeks after the CAR-T therapy, the patient achieved stringent complete response (sCR), and there were no PCs found in the morphology of bone marrow and peripheral blood with no abnormal PCs through FCM. Two weeks later, the morphology of BM plasma cells accounted for 2.5%, with FCM showing no abnormal PCs. Subsequently, repeated BM aspirates indicated sustained sCR. In November 2020, she was advised to take Venetoclax 10 mg per day due to a slightly higher λ FLC concentration. At that time, the free light chain ratio (κ/λ, FLCr) dropped from 0.9259 to 0.1884, with a reference range of 0.26–1.65. She had regular outpatient reexaminations at intervals of 1 to 3 months. In June 2021, the λ light chain level was 277.5 mg/L, in which time there was a >100 mg/L increase in the difference between λ and κ FLC levels. The condition relapsed again. One month later, an ultrasound showed that there were masses at the right neck and right adrenal gland pole. Biopsy and FCM of the right adrenal gland mass confirmed extramedullary plasmacytoma. There was no plasma cell seen in the peripheral blood at that time. The patient underwent regular anti-myeloma chemotherapies.

## Discussion

This patient with PCL secondary to MM showed rapid disease progression after five lines of therapy including PIs, an IMiD, daratumumab, and cytotoxic drugs, and was salvaged by CAR-T therapy. Dramatically, this sPCL patient obtained a progression-free survival (PFS) of 16 months after CAR-T therapy, which, to our knowledge, was the longest PFS for an sPCL patient treated with CAR-T therapy.

CRS cannot be decisively differentiated from infection in most cases. Actually, CRS and infection are closely intertwined because CRS increases the risk for infections, and infections increase the risk for CRS. There may be some overlap between the CRS and infection in some cases. Luo et al. propose a “double peaks of IL-6” pattern as a feature of life-threatening infection during the first 30 days post-CART infusion, in which the first increase appears during the CRS period and the second increase takes place during the period of severe infection (8). Similarly, as shown in **Figure 2**, this patient underwent a second increase in temperature, CRP, PCT, and inflammatory markers IL-6, IL-10, IFNγ, and TNFα, mainly due to a severe infection.

As is known to us all, PCL is a rare and highly aggressive clonal plasma cell dyscrasia characterized by the presence of CPCs (9, 10). Even in the era of novel agents, the prognosis of primary PCL (pPCL) remains unsatisfactory; 17.9%–45% achieved a complete response (CR) after the first-line treatment based on novel agents (11–14). The median overall survival (OS) is 1–2 years in the elderly, and almost 3 years in patients undergoing stem cell transplantation (SCT) (3, 10). The median PFS of pPCL patients is 5.5–12 months in the era of novel agents, and 20 months after receiving regimens combining bortezomib and an IMiD (thalidomide or lenalidomide) in the upfront treatment (5, 11–14). Furthermore, ASCT and maintenance therapy would prolong the PFS (11, 13). Allogeneic SCT cannot offer a significant survival advantage over autologous SCT (6, 11, 12). The prognosis of sPCL is much poorer, with a median OS of 4.0–4.2 months and a 19% 1-year OS rate (4, 14). Bortezomib-based regimens can prolong the OS of sPCL patients for about 3 months (14). After the daratumumab-based treatment, the median PFS and OS of sPCL patients are 2.5 and 5 months, respectively (15). Even lacking more data, it seems that the current therapies appear to be rarely effective against sPCL. Newer approaches under investigation including CAR-T therapy may emerge as a possible future therapeutic option for PCL patients.

However, there are few reports of CAR-T therapy treating sPCL patients, because the sPCL patients are usually excluded from the clinical trials (16–19). Nowadays, documentation on the use of CAR-T therapy in PCL is scarce (20, 21). In a phase I study, Zhou et al. found that the pPCL patients treated with anti-BCMA CAR T cell therapy had a PFS of 117–307 days, in which the CAR construct was composed of a murine anti-BCMA single-chain variable fragment (scFv), a CD8a hinge, the CD28 costimulatory molecule, and the intracellular signaling domain of CD3ζ (20). In another study, Zhou et al. developed a fully human BCMA-specific CAR (CT103A) with a fully human scFv, a CD8a hinge, and transmembrane domain, 4-1BB costimulatory, and CD3ζ activation domains. An RRMM patient who had extramedullary myeloma and developed sPCL received CT103A; the PFS and OS were 122 and 225 days, respectively, with the best response of VGPR (21, 22). In our study, the CAR is a second-generation BCMA-targeted CAR comprising 4-1BB costimulatory and CD3ζ signaling domains. After the CAR-T therapy, the patient obtained a 16-month PFS and 27-month OS with the best response of sCR. In addition, several studies also suggest that 4-1BB co-stimulation may be superior to CD28 in the context of CAR T cells (22).

CAR-T cell therapy has remarkably changed the treatment landscape of B-cell malignant tumors, encouraging further development in RRMM (23). In particular, anti-BCMA CAR-T therapy has established quite promising results in heavily pre-treated MM patients. The median PFS of patients treated with anti-BCMA CAR-T therapy was 2–15 months (19, 24, 25). The patients with some specific characteristics appear to benefit a lot from CAR-T therapy. However, it is quite difficult for us to recognize them. Moreover, CAR-T cells induce remission in RRMM patients when administered after one single cycle of lymphodepleting chemotherapy. It is important to increase the durability of response.

This case shows that sPCL patients can achieve a significant benefit after CAR-T therapy, which shows a promising future to extend the application to more PCL patients including pPCL. Moreover, CAR-T therapy is currently applied to the RRMM patients with previous exposure to PIs, IMiDs, CD38-targeted therapy, and ASCT or not a candidate for ASCT (16, 18). Applying CAR-T therapy as a frontline treatment deserves further exploration.

In conclusion, this case suggests that CAR-T therapy can effectively decrease tumor loads, improve the disease condition of sPCL patients, and potentially prolong patient survival. However, more studies are needed to carefully evaluate the benefit of anti-BCMA CAR-T therapy in PCL patients.

## Data availability statement

The raw data supporting the conclusions of this article will be made available by the authors, without undue reservation.

## Ethics statement

The studies involving human participants were reviewed and approved by Beijing Boren Hospital Ethics Committee. The patients/participants provided their written informed consent to participate in this study.

## Author contributions

WC and WG contributed to the conception of the study. JD wrote the first draft of the manuscript. WG revised the manuscript. YL, DZ, CT, and AC contributed significantly to analysis, visualization, and manuscript preparation. All authors contributed to the article and approved the submitted version.

## Funding

This project was supported by the Beijing Natural Science Foundation (7212041) and the China Medical Education Association (2020KTE009).

## Acknowledgments

The authors would like to thank the participating centers and members.

## Conflict of interest

AC is a founding member of Shanghai YaKe Biotechnology Ltd., a biotechnology company focusing on research and development of tumor cellular immunotherapy.

The remaining authors declare that the research was conducted in the absence of any commercial or financial relationships that could be construed as a potential conflict of interest.

## Publisher’s note

All claims expressed in this article are solely those of the authors and do not necessarily represent those of their affiliated organizations, or those of the publisher, the editors and the reviewers. Any product that may be evaluated in this article, or claim that may be made by its manufacturer, is not guaranteed or endorsed by the publisher.
