# Supplementary material for: Case report: Plasma cell leukemia secondary to multiple myeloma successfully treated with anti-BCMA CAR-T cell therapy
Source: Front Oncol. 2022 Sep 21;12:901266. doi: 10.3389/fonc.2022.901266 (PMC9533140; doi:10.3389/fonc.2022.901266)
Supplement: Supplementary file 1 [file Table_1.docx]

Supplementary Material

# Table 1 Summary of treatment and response for the RRMM patient

| Line of therapy | Regimen | Best Response | duration of response (months) |
| --- | --- | --- | --- |
| 1st | BD, BCD | PR | 7 |
| 2nd | DCD, CD, Dex | PR | 20 |
| 3rd | Rd | VGPR | 18 |
| 4th | ICD, IRCD | PR | 12 |
| 5th | BBDD, DVD | PR | 2 |
| 6th | DVRD, CAR-T therapy | sCR | 16 |
| 7th | XPD | SD | 4 |
| 8th | VCAD, VRCDD, VCDD, radiotherapy | PR | 6(until April,2022) |

PR: partial response; VGPR: very good partial response; sCR: stringent complete response; SD: stable disease; BD: bortezomib, dexamethasone; BCD: bortezomib, cyclophosphamide, dexamethasone; DCD: liposomal doxorubicin, cyclophosphamide, dexamethasone; CD: cyclophosphamide, dexamethasone; Dex: dexamethasone; Rd: lenalidomide, dexamethasone; ICD: ixazomib, cyclophosphamide, dexamethasone; IRCD: ixazomib, lenalidomide, cyclophosphamide, dexamethasone; BBDD: bendamustine, bortezomib, daratumumab, dexamethasone; DVD: daratumumab, bortezomib, dexamethasone; DVRD: daratumumab, bortezomib, lenalidomide, dexamethasone; XPD: selinexor, pomalidomide, dexamethasone; VCAD: bortezomib, cyclophosphamide, epirubicin, dexamethasone; VRCDD: bortezomib, lenalidomide, cyclophosphamide, liposomal doxorubicin, dexamethasone; VCDD: bortezomib, cyclophosphamide, liposomal doxorubicin, dexamethasone.
